# Complete Genome Sequence of the Itraconazole Decreased Susceptible *Madurella fahalii* Type-Strain CBS 129176

**DOI:** 10.1007/s11046-023-00807-0

**Published:** 2024-01-17

**Authors:** Mickey Konings, Bert Gerrits van den Ende, Mirthe W. J. Raats, Ahmed Hassan Fahal, Wendy W. J. van de Sande, Ferry Hagen

**Affiliations:** 1 Department of Medical Microbiology and Infectious Diseases, Erasmus MC, University Medical Center Rotterdam, PO Box 2040, 3000 CA Rotterdam, The Netherlands; 2 Department of Medical Mycology, Westerdijk Fungal Biodiversity Institute, Utrecht, The Netherlands; 3 The Mycetoma Research Center, University of Khartoum, Khartoum, Sudan; 4 Institute for Biodiversity and Ecosystems Dynamics, University of Amsterdam, Amsterdam, The Netherlands; 5 Department of Medical Microbiology, University Medical Center Utrecht, Utrecht, The Netherlands

**Keywords:** *Madurella mycetomatis*, *Madurella fahalii*, Nanopore sequencing, De novo genome assembly, Decreased itraconazole susceptibility

## Abstract

*Madurella fahalii* is a causative agent of the implantation mycosis mycetoma with decreased susceptibility to itraconazole, the preferred therapeutic drug to combat mycetoma. Here, we report the *M. fahalii* type-strain CBS 129176 genome assembly and annotation to identify a glutamic acid insert near the azole-binding pocket in the Cyp51A protein.

## MycopathologiaGENOME

*Madurella fahalii* is one of the four species currently within the genus *Madurella* [1]*.* All species within this genus are causative agents of human mycetoma, a neglected tropical disease characterized by subcutaneous tumorous lesions. A characteristic of this infection is that the causative agents organize themselves in grains. In the case of *Madurella* species these grains are black. *Madurella mycetomatis* is by far the most common. A decade ago, the three species, *Madurella fahalii, M. tropicana* and *M. pseudomycetomatis,* were described [2]. *Madurella* species share similar morphology and are non-sporulating. They can only be differentiated to species level by molecular identification methods [3]. These molecular tools are not widely available in endemic regions and as a result the epidemiology of the different *Madurella* species remains widely unknown [4, 5]. The feature which makes *M. fahalii* different from its sibling species, is that all the currently described isolates have decreased susceptibility to itraconazole, the current drug of choice for mycetoma therapy [2, 6]. The molecular mechanisms behind this decreased susceptibility remain enigmatic.

Therefore, in order to improve design of molecular identification tools and uncover the mechanism of decreased susceptibility, we extracted high-quality genomic DNA of *M. fahalii* type-strain CBS 129176 as previously described [7]. This type-strain was originally isolated in September 1999 at the WHO collaborative Mycetoma Research Center in Khartoum, Sudan, from a 45-year-old male from Omdurman with a large mycetoma lesion (> 10 cm in diameter) on his left sole [2]. Long-read nanopore sequencing was performed on the DNA using the ligation sequencing library preparation kit (SQK-LSK114.24; ONT, Oxford, UK), followed by sequencing the library onto a MinION flow cell (FLO-MIN114; ONT) as described by the manufacturer. Guppy v6.4.2 (ONT) was used to basecall the raw data in the high-accuracy mode, thereafter de novo genome assembly was carried out using Flye v2.9 and resulted in 8 fragments representing 7 chromosomes (total 39,039,837 bp, range 9,419,784–2,207,012 bp) and the mitochondrial genome (40,076 bp) that had 55X and 1322X coverage, respectively. The assembled genome had an N50 of 5,590,309 bp and a GC-content of 54.8%. Genome annotation was performed using the Funannotate pipeline v1.8.15 (https://github.com/nextgenusfs/funannotate) and resulted in an annotated genome that contains 10,921 predicted genes, 10,734 mRNAs, and 187 tRNAs, also 480 CAZymes and 319 proteases were predicted. (BioProject PRJNA913940, BioSample SAMN32314170, Sequence Read Archive SRR22816638, and Genome accession number JAPYLN000000000) [8].

**Fig. 1:**
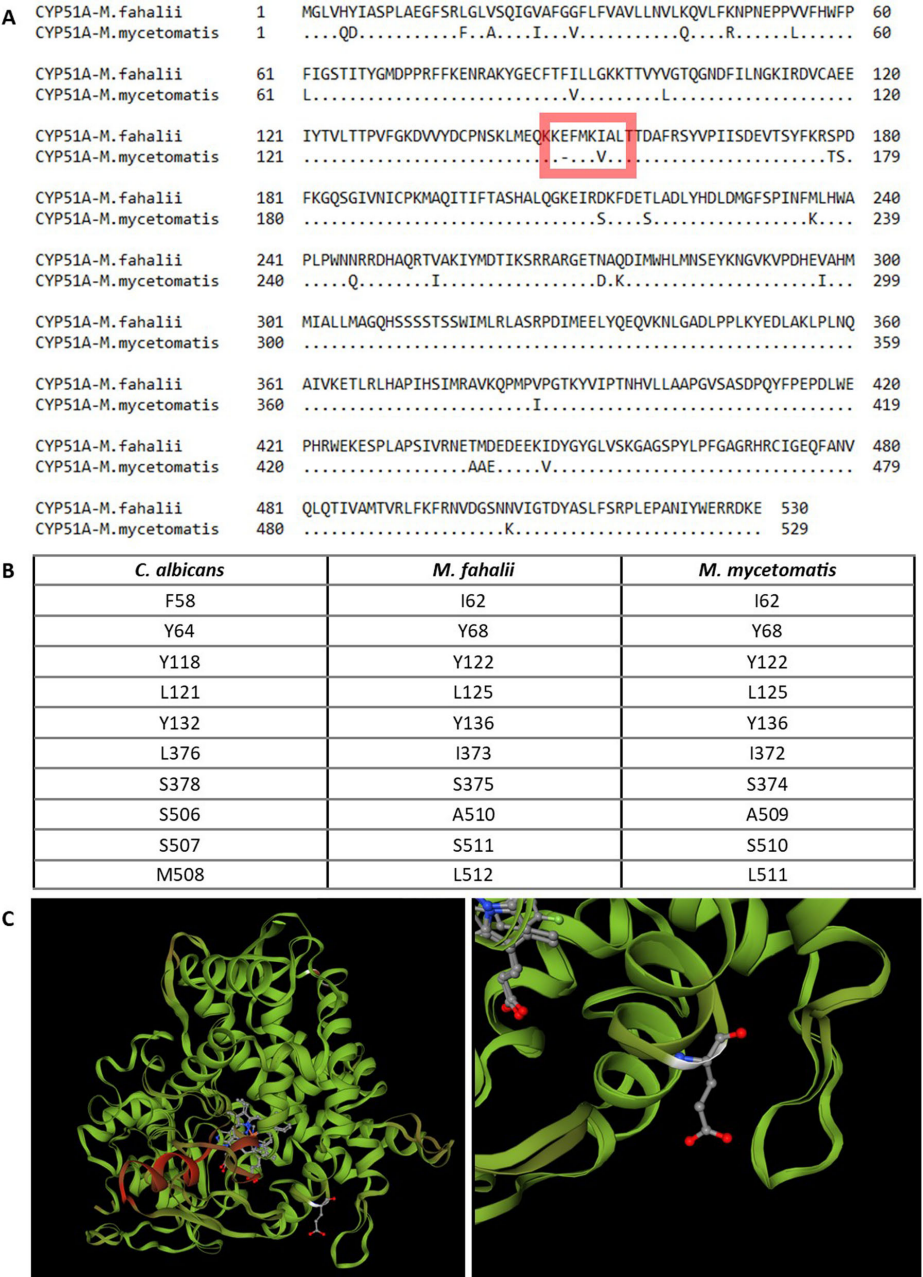
**a** Alignment of the eburicol 14-α-demethylase (Cyp51A) protein sequences originating from *M. fahalii* (OQ566866) and *M. mycetomatis* (UVX19995.1)*.* The protein sequence of *M. fahalii* contains in total 30 amino acid variations, of which one additional amino acid compared to the respective *M. mycetomatis* Cyp51A protein. **b** Comparison of the key residues for the binding of itraconazole to the Erg11 protein (Cyp51A orthologue in *C. albicans*) to the respective residues in *M. fahalii* and *M. mycetomatis* [9]*.* **c** Predicted 3D model comparison of the Cyp51A protein of both *M. fahalii* and *M. mycetomatis*. The protein models are visualized as overlapping structures. The region highlighted in red on the left panel indicates low confidence in the predicted 3D model. The region of interest is highlighted on the right panel, displaying the glutamic acid insert present in the sequence of *M. fahalii*

In order to determine if a difference in the drug target of the azoles could be responsible for the decreased susceptibility of *M. fahalii* towards itraconazole, the *M. fahalii CYP51A* sequence was obtained from the genome sequence and compared to that of *M. mycetomatis* strain MM55 (BioProject PRJNA267680, KXX80456.1). Using the standard in vitro susceptibility testing assay for *Madurella* species the minimal inhibitory concentration of itraconazole was reported as > 16 µg/mL for *M. fahalii* CBS 129176 and 0.06 µg/mL for *M. mycetomatis* MM55 [2]. The translated coding sequences of *CYP51A* (Fig. 1A) were aligned and compared using NCBI protein BLAST and MEGA-X. Thirty amino acid variations were observed between *M. fahalii* and *M. mycetomatis* (Fig. 1a). The key residues for binding of itraconazole as identified for *ERG11* (*CYP51A* orthologue) in *C. albicans* (BioProject PRJNA14005, XP_716761.1), were compared against the respective residues in both *M. fahalii* and *M. mycetomatis* using MEGA-X [9]. Comparing the key residues involved in binding of itraconazole based on *C. albicans ERG11*, no differences were found between the respective residues (Fig. 1b). However, the insert of glutamic acid observed on position 149 and the shift from isoleucine to valine on position 153 were in a region associated with azole resistance in the *Candida albicans* homologue of this gene [10, 11] ^1,2^.

Cyp51A 3D structure models were generated using SWISS-MODEL with the *Aspergillus fumigatus* crystal structure of Cyp51B as template [12, 13]. The generated structures were aligned using the built-in function of SWISS-MODEL. The structure alignment of the predicted Cyp51A 3D models for *M. fahalii* and *M. mycetomatis* mainly display a structural discrepancy at the site of the glutamic acid insert, which is near the expected azole-binding site (Fig. 1c). Although this insertion therefore is the most likely residue linked to the decreased susceptibility to itraconazole, further research is required to provide definite validation for the decreased susceptibility of *M. fahalii*.

## References

[1] Ahmed Sarah A, de Hoog GS, van de Sande Wendy WJ. Fungi causing eumycotic mycetoma*.* 2019. 10.1128/9781683670438.MCM.ch128

[2] de Hoog GS, van Diepeningen AD, el Mahgoub S, van de Sande WW (2012). New species of *Madurella*, causative agents of black-grain mycetoma. J Clin Microbiol.

[3] Arastehfar A, Lim W, Daneshnia F (2020). *Madurella* real-time PCR, a novel approach for eumycetoma diagnosis. PLoS Negl Trop Dis.

[4] Ahmed E, Nour B, Abakar A (2020). The genus *Madurella*: Molecular identification and epidemiology in Sudan. PLoS Negl Trop Dis.

[5] van de Sande WWJ, Fahal AH, Goodfellow M, Mahgoub ES, Welsh O, Zijlstra EE (2014). Merits and pitfalls of currently used diagnostic tools in mMycetoma. PLoS Negl Trop Dis.

[6] Nyuykonge B, Lim W, van Amelsvoort L (2022). Eumycetoma causative agents are inhibited in vitro by luliconazole, lanoconazole and ravuconazole. Mycoses.

[7] Navarro-Muñoz JC, de Jong AW, van den Ende BG (2019). The high-quality complete genome sequence of the opportunistic fungal pathogen *Candida vulturna* CBS 14366T. Mycopathologia.

[8] Kolmogorov M, Yuan J, Lin Y, Pevzner PA (2019). Assembly of long, error-prone reads using repeat graphs. Nat Biotechnol.

[9] Shi N, Zheng Q, Zhang H (2020). Molecular dynamics investigations of binding mechanism for triazoles inhibitors to *CYP51*. Front Mol Biosci.

[10] Stephanie AF, Brendan C, Sarah GW, Mary AS, Rogers PD (2015). Contribution of clinically derived mutations in *ERG11* to azole resistance in *Candida albicans*. Antimicrob Agents Chemother.

[11] UniProt C. P10613 CP51_CANAL. https://www.uniprot.org/uniprotkb/P10613/entry. Accessed 31–01–2023.

[12] Waterhouse A, Bertoni M, Bienert S (2018). SWISS-MODEL: Homology modelling of protein structures and complexes. Nucleic Acids Res.

[13] Hargrove TY, Wawrzak Z, Lamb DC, Guengerich FP, Lepesheva GI (2015). Structure-functional characterization of cytochrome P450 sterol 14α-demethylase (*CYP51B*) from *Aspergillus fumigatus* and molecular basis for the development of antifungal drugs. J Biol Chem.

